# Low-Grade Inflammation Is Associated with Apathy Indirectly via Deep White Matter Lesions in Community-Dwelling Older Adults: The Sefuri Study

**DOI:** 10.3390/ijms20081905

**Published:** 2019-04-17

**Authors:** Hiroshi Yao, Yoshito Mizoguchi, Akira Monji, Yusuke Yakushiji, Yuki Takashima, Akira Uchino, Takefumi Yuzuriha, Manabu Hashimoto

**Affiliations:** 1 Division of Clinical Research, National Hospital Organization Hizen Psychiatric Center, Saga 842-0192, Japan; ytakahizen@yahoo.co.jp (Y.T.); takefumi@hosp.go.jp (T.Y.); ma9hashi@yahoo.co.jp (M.H.); 2 Department of Psychiatry, Faculty of Medicine, Saga University, Saga 849-8501, Japan; ymizo@cc.saga-u.ac.jp (Y.M.); amonji@hf.rim.or.jp (A.M.); 3 Division of Neurology, Department of Internal Medicine, Faculty of Medicine, Saga University, Saga 849-8501, Japan; yakushij@cc.saga-u.ac.jp; 4 Department of Diagnostic Radiology, Saitama Medical University International Medical Center, Saitama 350-1298, Japan; auchino@saitama-med.ac.jp

**Keywords:** physical activity, apathy, vascular depression, vascular cognitive impairment, white matter lesions, magnetic resonance imaging, small vessel disease, silent stroke

## Abstract

Low-grade inflammation is implicated in the pathogenesis of atherosclerosis, metabolic syndrome, and apathy as a form of vascular depression. We analyzed the brain magnetic resonance imaging findings in 259 community-dwelling older adults (122 men and 137 women, with a mean age of 68.4 years). The serum concentrations of high-sensitivity C-reactive protein (hsCRP) were measured by a quantitative enzyme-linked immunosorbent assay. Logistic regression analysis revealed that the log_10_ hsCRP value and the presence of a metabolic syndrome were independently associated with confluent but not punctate deep white matter lesions (DWMLs). Path analysis based on structural equation modeling (SEM) indicated that the direct path from the log_10_ hsCRP to the DWMLs was significant (β = 0.119, *p* = 0.039). The direct paths from the metabolic syndrome to the log_10_ hsCRP and to the DWMLs were also significant. The direct path from the DWMLs to apathy (β = −0.165, *p* = 0.007) was significant, but the direct path from the log_10_ hsCRP to apathy was not significant. Inflammation (i.e., elevated serum hsCRP levels) was associated with DWMLs independent of common vascular risk factors, while DWMLs were associated with apathy. The present analysis with SEM revealed the more realistic scheme that low-grade inflammation was associated with apathy indirectly via DWMLs in community-dwelling older adults.

## 1. Introduction

Low-grade inflammation, which is typically determined by increased levels of high-sensitivity C-reactive protein (hsCRP), has been recognized in several studies as a risk factor for ischemic stroke, independent of other cardiovascular risk factors [1,2,3,4]. However, one study reported contrasting results [5]. Higher levels of CRP (C-reactive protein), but not interleukin-6 (IL-6) [6] or the CRP level relative to that of IL-6 [7], were associated with increased risk of ischemic stroke in population-based studies. An earlier study reported that higher levels of both CRP and IL-6 were associated with silent brain infarction even when adjusted with traditional vascular risk factors [8], whereas higher CRP levels were associated with the presence and progression of deep white matter lesions (DWMLs) [9,10] or white matter integrity [11] rather than lacunar infarcts. Although one study found that CRP was not associated with small vessel disease-related brain lesions (i.e., the volume of white matter lesions and the number of lacunes) [12], cerebral small vessel disease and particularly DWMLs—the predominant basis of vascular cognitive impairment [13]—may be partly caused by systemic inflammation in addition to classical vascular risk factors.

Initially, the role of CRP in mood disorders was intriguing but somewhat ambiguous partly due to study design (e.g., case-control studies) and insufficient adjustment for potential confounders [14]. Recent meta-analyses revealed that elevated levels of inflammation markers were found to be associated with an increased risk of depression in the general population [15,16]. However, this association could be confounded by the symptoms of apathy, that is, apathy and depression might be confused due to overlapping clinical features. Although apathy is one of the symptoms of depression, apathy (a lack of motivation without depressed mood, guilt, and hopelessness) can also be discriminated from depression [17,18]. A population-based cohort study indicated that increased CRP levels were associated with apathy symptoms but not with depressive symptoms [17]. Furthermore, apathy rather than depression may be more closely related to cardiovascular risk factors and cardiovascular disease [18]. Several studies, including ours, have reported that ‘silent’ ischemic brain lesions such as DWMLs [19,20,21,22,23] or lacunar infarcts in DWMLs [24,25] contributed to depression or apathy in healthy older adults, supporting the concept of the vascular depression hypothesis [26]. Therefore, we hypothesized that apathy would be associated with systemic inflammation indirectly via DWMLs.

## 2. Results

### 2.1. Background Characteristics

For the 259 subjects (122 men and 137 women, with a mean age of 68.4 years) enrolled in this study, the mean education duration (years of school) was 11.5 years. None of the subjects included in the study had clinical depression, while 24 (9.3%) reported having a depressed mood. Silent brain infarction, DWMLs, periventricular hyperintensities, and cerebral microbleeds were detected in 26 (10.0%), 90 (34.7%), 40 (15.4%), and 19 (7.3%) subjects, respectively. The numbers of subjects with DWMLs by grade were as follows: grade 0 (*n* = 169), grade 1 (*n* = 61), grade 2 (*n* = 28), and grade 3 (*n* = 1).

Although the hsCRP level distribution was highly skewed (median: 0.589 mg/L, interquartile range: 0.292-1.326 mg/L), the log-transformed hsCRP values (log_10_ hsCRP) were normally distributed (Shapiro–Wilk test, *p* = 0.362). The mean log_10_ hsCRP value in the grades 2–3 (confluent) DWMLs group was significantly higher than those in the grade 0 and grade 1 DWMLs group (*p* = 0.018 and *p* = 0.050, respectively). The mean of the apathy scale scores in the grades 2–3 (confluent) DWMLs group was 414 ± 123, which was significantly lower (more apathetic) than that in the grades 0–1 DWMLs group (478 ± 110, *p* = 0.014). The characteristics of the study population among tertiles of hsCRP values are provided in Table 1. Medium to high tertiles of CRP were associated with metabolic syndrome and its components (waist circumference, triglyceride, and high-density lipoprotein (HDL) cholesterol), body mass index, and uric acid. We produced a metabolic syndrome score as the sum of the four essential components of metabolic syndrome (i.e., waist circumference, blood pressure, triglyceride and/or HDL cholesterol, and blood glucose). The log_10_ hsCRP value was correlated with metabolic syndrome score, and an analysis of variance (ANOVA) revealed that the log_10_ hsCRP value in the score 4 group was significantly higher than that in the score 0 group after post hoc Bonferroni testing (*p* = 0.038). The leukocyte count was significantly correlated with the log_10_ hsCRP value (*r* = 0.28, *p* < 0.001).

Because apathy may affect cognitive function, we analyzed the association between cognitive function and apathy. Although the modified Stroop Test (mST) was significantly associated with the apathy scale (Pearson’s correlation, *r* = −0.141, *p* = 0.025), executive dysfunction, determined by the most prolonged quintile of mST, was not associated with apathy scale in univariate logistic regression analysis. Memory dysfunction, determined by the lowest quintile of the Rivermead Behavioral Memory Test (RBMT, standard profile score), was significantly associated with the apathy scale (odds ratio (OR) = 0.965; 95% confidence interval (CI), 0.939–0.992; *p* = 0.012), this association was no longer significant after adjustment for age, sex, education, and hippocampal atrophy. The independent predictors of memory dysfunction, defined by the RBMT < 17 (54 of 256 [21.1%]), were age (OR per 10 years 2.486, 95% CI, 1.542–4.007, *p* < 0.001) and hippocampal atrophy (OR per 1-*z*-score 4.163, 95% CI, 1.825–9.492, *p* = 0.001). Taken together, a direct association of apathy with cognitive function was unlikely as far as we could investigate.

### 2.2. Logistic Regression Analysis

Multivariate analysis was carried out with logistic regression analysis for DWMLs as the dependent variable and the log_10_ hsCRP value, age, sex, hypertension, diabetes mellitus, hyperlipidemia, renal function, metabolic syndrome, uric acid, alcohol use, and smoking as the independent variables. When possible confounders, including vascular risk factors and the log_10_ hsCRP value, were entered into the binary logistic regression model (the forward stepwise method), the independent predictors of DWMLs were age (OR = 2.456/10 years; 95% CI, 1.647–3.663; *p* < 0.001) and hypertension (OR = 2.620; 95% CI, 1.508–4.553; *p* = 0.001), while the independent predictors of confluent DWMLs were age (OR = 4.405; 95% CI, 2.419-8.021; *p* < 0.001), the log_10_ hsCRP value (OR = 3.024; 95% CI, 1.305–7.008; *p* = 0.010) and metabolic syndrome (OR = 3.211; 95% CI, 1.141–9.003; *p* = 0.027) (Table 2). When the presence of metabolic syndrome (Model 1) was replaced by the metabolic syndrome score (Model 2), the associations between confluent DWMLs and age, log_10_ hsCRP, and the metabolic syndrome (score) were essentially the same.

### 2.3. Structural Equation Modeling

**Figure 1:**
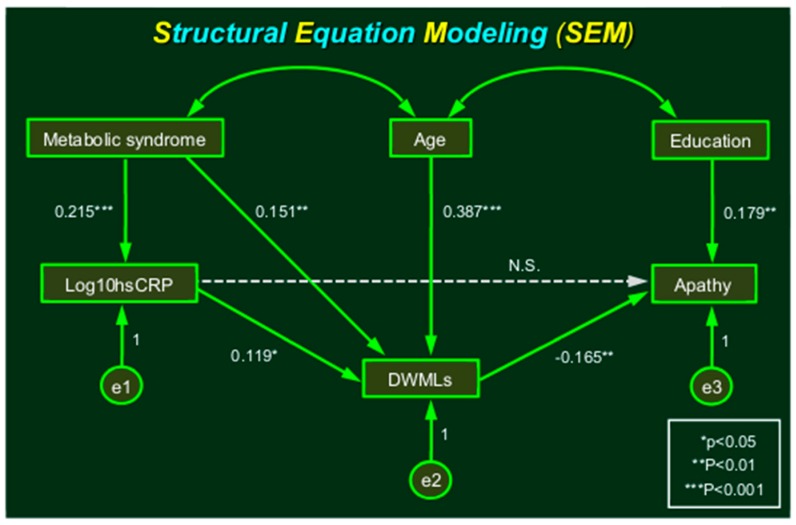
Structural equation modeling (SEM) shows that apathy was indirectly associated with inflammation (log_10_ hsCRP) through deep white matter lesions (DWMLs).

These findings mentioned above lead us to the hypothesis that metabolic syndrome and inflammation would cause DWMLs, and that DWMLs might intensify apathetic behavior. We investigated the relationship between metabolic syndrome, the log_10_ hsCRP value, DWMLs, and apathy, using a graphical multivariate analysis structural equation modeling (SEM). Apathy was quantified with a visual analog version of the Starkstein’s apathy scale [22]. Depressed mood was defined as being always or frequently present for this symptom. The SEM was described as path diagrams, where the square boxes represented measured observations, and circles represented latent constructs. Single-headed arrows represented a simple regression relationship, and double-headed arrows represented correlations (Figure 1). Path analysis based on SEM indicated that the direct path from log_10_ hsCRP to DWMLs was significant (β = 0.119, *p* = 0.039). The direct paths from the metabolic syndrome (score) to log_10_ hsCRP and to DWMLs were also significant. The direct path from DWMLs to apathy was significant (β = −0.165, *p* = 0.007), as was the direct path from education to apathy (β = 0.179, *p* = 0.003). However, the direct path from log_10_ hsCRP to apathy was not significant. When apathy was replaced by depressed mood, the direct paths from DWMLs or education to depressed mood were no longer significant. Therefore, apathy but not depressed mood was indirectly associated with inflammation (i.e., log_10_ hsCRP) via DWMLs. The measures of model fitness were as follows: chi-square N.S., goodness of fit index (GFI) = 0.993, adjusted goodness of fit index (AGFI) = 0.979, comparative fit index (CFI) = 1.000, and root mean square error of approximation (RMSEA) = 0.000. Thus, the presented model reasonably fit the data.

## 3. Discussion

In the present cross-sectional study, we observed that the presence of confluent but not punctate DWMLs was associated with metabolic syndrome and low-grade inflammation. Silent brain infarction (i.e., lacunes as the prototype of small vessel disease) was not associated with inflammation, suggesting that moderate- to high-grade DWMLs may not be the consequence of ‘pure’ small vessel disease. SEM analysis revealed that metabolic syndrome might be one of the risk factors for DWMLs directly and indirectly via inflammation. Although apathy was associated with the presence of DWMLs, a direct link between inflammation and apathy was not indicated.

The term subcortical ischemic vascular disease is often used for both lacunar infarction and white matter lesions, but the pathophysiology may differ; deep white matter is particularly vulnerable to brain hypoperfusion [27]. The medullary and perforating arteries are end arteries, thus making the periventricular border zones the most susceptible to decreased cerebral blood flow (CBF) [28,29]. Typically, no significant stenosis of the extracranial or intracranial arteries is observed in Binswanger’s disease or extensive DWMLs [30,31], suggesting that extensive DWMLs may be caused by compromised CBF without vascular occlusion. Decreased CBF with a transient rise in the oxygen extraction fraction (i.e., misery perfusion) [32,33] or low baseline CBF [34] was observed in association with DWMLs, thereby indicating the ischemic origin of DWMLs. Taken together, DWMLs are considered to be a ‘hybrid’ of small and large vessel disease.

Arteriosclerosis affects the deep basal ganglia, the brainstem and white matter perforator vessels, thereby causing cerebral small vessel disease [35]. In contrast, atherosclerosis is an inflammatory disease of the large- and medium-sized artery walls, characterized by complex immune activations (Supplementary Figure S1) [36,37,38]. In brief, retention and oxidation of low-density lipoprotein (LDL) particles in an artery induces pro-inflammatory responses. Adhesion and migration of monocytes/macrophages, augmented by leukocyte adhesion molecules on the endothelial cells, results in the uptake of oxidized LDL, which leads to the formation of foam cells. Furthermore, activated macrophages and T cell lymphocytes are involved in the advanced atherosclerotic lesion. Endothelial dysfunction characterized by increased endothelin-1 and decreased nitric oxide is also observed. Furthermore, oxidized LDLs increase the expression of growth factors such as platelet-derived growth factor and fibroblast growth factor for the migration and proliferation of smooth muscle cells, leading to the thickening of the plaques, formation of the necrotic core, and ultimately plaque rupture. As a consequence of these complex immune responses, the majority of circulating CRP is produced by hepatocytes under the regulatory control of circulating IL-6 [36,37,38].

Low grade inflammation is characteristic of the metabolic syndrome, as hsCRP levels have been found to correlate with the key components of metabolic syndrome, such as elevated triglyceride, low HDL cholesterol, central obesity, elevated blood pressure, and high fasting glucose levels or insulin resistance [39,40]. In the metabolic syndrome, the concentrations of pro-inflammatory cytokines (e.g., IL-6 and tumor necrosis factor-α [TNF-α]), markers of pro-oxidant status (e.g., oxidized LDL and uric acid), and prothrombotic factors were elevated, while the levels of anti-inflammatory cytokines (e.g., IL-10), ghrelin, adiponectin, and antioxidant factors were decreased [41]. The inflammatory process that occurs in obese people differs from the classical inflammatory response; the toll-like receptor 4 signaling pathway, which is activated by saturated fatty acids, has been acknowledged as one of the main triggers of the obesity-induced inflammation [42]. Previous studies have reported that metabolic syndrome may exert its detrimental effects on silent ischemic brain lesions, particularly DWMLs, on magnetic resonance imaging (MRI) via inflammation in addition to classical risk factors [43,44].

Our study possesses several limitations of note, including its cross-sectional study design, which limits the interpretation of our results with respect to cause and effect. Although it is generally, but not fully, accepted that inflammation is one of the causes of DWMLs, as discussed in the Introduction section, we cannot definitively conclude as such based on our present results. Another limitation is that CRP is the downstream event of inflammation; therefore, the precise mechanisms of damage involved in the formation of DWMLs are unclear. In addition, we cannot exclude the possibility of residual confounding related to factors not included in the SEM analysis. However, in addition to the first-generation regression-based approaches, SEM revealed the more realistic scheme that apathy might be associated with inflammation indirectly, via DWMLs. Another strength of our study includes the use of MRI in community-dwelling subjects. For example, the incidence of silent brain infarction is five times higher than that of strokes in the general population, and most cardiovascular risk factors increase the risk of silent ischemic lesions on MRI [45], indicating that silent ischemic lesions on MRI are better surrogate markers of cerebral arteriosclerosis than symptomatic strokes.

In conclusion, inflammation and metabolic syndrome were independently associated with the presence of confluent but not punctate DWMLs. Path analysis based on SEM indicated that the direct paths from log_10_ hsCRP and metabolic syndrome to DWMLs were significant. Although the direct path from DWMLs to apathy was significant, the direct path from log_10_ hsCRP to apathy was not significant. Therefore, inflammation was associated with apathy indirectly via DWMLs in healthy older adults. Recently, the CANTOS trial indicated that directly reducing inflammation with an IL-1β-neutralizing monoclonal antibody could reduce the rate of cardiovascular events [46,47]. In future studies, investigation further upstream in the inflammatory cascade from CRP and IL-6 to IL-1 may provide novel targets for protection against DWMLs.

## 4. Subjects and Methods

### 4.1. Participants and Protocol Approval

Between 2010 and 2016, we performed a cross-sectional observational study in the rural community of Sefuri village (Saga, Japan), which had a total population of 1739 people as of April 2014. We examined consecutive 297 volunteers aged 60–89 years, who were independent in their daily life without apparent dementia. A total of 38 subjects were excluded due to cognitive impairment (*n* = 7); psychiatric disorders, including depression (*n* = 5); claustrophobia or contraindications for MRI (*n* = 8); a history of stroke (*n* = 9); brain tumor (*n* = 1); chronic subdural hematoma (*n* = 1); a history of head trauma (*n* = 3); chronic renal failure (*n* = 3); and insufficient clinical information (*n* = 1). Finally, we analyzed 259 subjects in the present study.

The National Hospital Organization Hizen Psychiatric Center Institutional Review Board approved the study (approval number: 15-1 and 24-4). Written informed consent was obtained by H.Y. from all participants.

### 4.2. Clinical Assessments

The participants underwent a structured clinical interview, general hematology tests including leukocyte count, and biochemical tests. Blood pressure was measured in the sitting position using the standard cuff method; beginning in 2013, simultaneous blood pressure measurements were recorded from both arms using a pair of automated sphygmomanometers (Omron model HEM-1020, Omron, Japan). The blood pressure values obtained from the right arm were used throughout the current study. Vascular risk factors were defined as previously described [48]. Briefly, arterial hypertension was considered to be present in participants with a history of repeated blood pressure recordings ≥140/90 mmHg, and in those being treated for hypertension. Diabetes mellitus was defined as a fasting plasma glucose level of ≥6.99 mmol/L (126 mg/dL) and/or HbA1c of ≥6.5%, or a previous diagnosis of diabetes mellitus. Hyperlipidemia was considered to be present in participants with a total serum cholesterol concentration of ≥5.69 mmol/L (220 mg/dL), and in those being treated for hyperlipidemia. Because the optimal cutoff points of waist circumference for predicting cardiovascular disease in Japan were 90 cm for men and 80 cm for women [49], central obesity was defined by waist circumferences of ≥90 cm for men and ≥80 cm for women in the present study. Metabolic syndrome was defined by the presence of central obesity and a minimum of two of three factors: a blood pressure of ≥130/85 mmHg, a fasting blood glucose level of ≥6.1 mmol/L (110 mg/dL) or blood glucose level of ≥7.77 mmol/L (140 mg/dL) in the case of non-fasting settings, and a triglyceride level of ≥1.69 mmol/L (150 mg/dL) and /or HDL cholesterol level of <1.03 mmol/L (40 mg/dL). We produced a metabolic syndrome score as the sum of the four indices defined above (i.e., waist circumference, blood pressure, triglyceride and /or HDL cholesterol, and blood glucose). The presence of each index above the threshold value was counted as 1 to generate total scores of between 0 and 4. The estimated glomerular filtration rate (eGFR) was calculated using the Modification of Diet in Renal Disease equation for the Japanese modification: eGFR (mL/min/1.73 m^2^) = 194 × (serum creatinine [mg/dL])^−1.094^ × (age)^−0.287^ × (0.739 if female) [50]. Smoking was defined as the participant smoking an average of at least 10 cigarettes per day, while former smokers were considered nonsmokers. Alcohol use was defined as the participant reporting drinking one or more alcoholic beverages (10 g of ethanol) per week.

### 4.3. Apathy Scale

Each item of the Starkstein’s apathy scale [51] was quantified on a visual analog scale, where one end of a 60 mm long line is ‘absolutely correct’ and the other end is ‘completely wrong’, as previously described [23,52]. Because the item–total correlations of questions 3 (Are you concerned about your condition?) and 11 (Are you unconcerned with many things?) had been weak out of the 14 original questions, we excluded the scores of these two questions from the analysis. This apathy scale yields total scores of 0–720, with lower scores indicating apathetic behavior. Depressed mood and insomnia were rated as ‘none’, ‘sometimes’, ‘frequent’, and ‘always’; depressed mood and insomnia were defined as an always or frequent presence of these symptoms. Patients who had been previously diagnosed with clinical depression or were taking medication for depression were excluded from the study.

Because apathy was associated with physical inactivity [23], and physical inactivity was associated with hippocampal atrophy and memory impairment [53], apathy may be directly or indirectly related to cognitive disturbance. Therefore, we analyzed the association between cognitive function tests and the apathy scale. Memory and executive function were assessed with RBMT and mST, respectively, as previously described [50,53]. As mST was not available in three cases, the relationship between apathy and cognitive function was examined in 256 subjects.

### 4.4. High-Sensitivity CRP Measurements

Serum specimens were stored at −80 °C until the measurements. We measured hsCRP using a quantitative enzyme-linked immunosorbent assay with a sensitivity of 25 ng/L (Human C-Reactive Protein/CRP Quantikine ELISA Kit, R&D Systems, Inc., Minneapolis, MN, USA). According to the manufacturer’s instructions, the samples were assayed in duplicate and the absorbance was measured at 450 nm. The final concentrations were calculated from the respective standard curves and expressed as mg/L.

### 4.5. Assessment of MRI Findings

A combination of T1-weighted, T2-weighted, and fluid attenuated inversion recovery images (FLAIR) is required to accurately detect both silent brain infarction and white matter lesions [54]. Imaging was performed on a 1.5T MRI scanner (Achieva, Philips, the Netherlands) using the T1- and T2-weighted, fluid-attenuated inversion recovery, and T2*-weighted images. Silent brain infarction was defined by low signal intensities on T1-weighted images, and high signal intensity areas on T2-weighted images, and a diameter of ≥3 mm, as previously described. We differentiated enlarged perivascular spaces from silent brain infarction based on their location, shape, and size. Lesions of <3 mm in diameter are more likely to be perivascular space than lacunes, and the presence of moderate to severe basal ganglia perivascular space was recorded [55]. The white matter lesions were defined as isointense with normal brain parenchyma on T1-weighted images, and high signal intensity areas on T2-weighted images. We used the validated rating scale of DWMLs by Fazekas et al.: grade 0, absent; grade 1, punctate foci; grade 2, beginning confluence of foci; and grade 3, large confluent areas [56]. For periventricular hyperintensities, we determined the presence and severity (grade 0, absent; grade 1, pencil thin; and grade 2, smooth halo lining) using FLAIR images. Two authors (H.Y. and A.U.), who were blinded to all clinical data, independently reviewed all scans. We evaluated the degree of hippocampal atrophy, using a free software program—the Voxel-Based Specific Regional Analysis System for Alzheimer’s Disease (VSRAD) advance version, as previously described [53].

### 4.6. Statistical Analysis

All clinical variables are presented as the mean ± standard deviation. All tests were two-sided, and the level of statistical significance was set at *p* < 0.05. The data were analyzed using IBM SPSS Statistics version 18 for Windows (SPSS Japan Inc., Tokyo, Japan). For the univariate analysis, the chi-square test or Fisher’s exact test were used to investigate between-group differences in categorical variables, while unpaired t-tests were used to investigate differences in continuous variables. Pearson’s correlation coefficients were used to assess the relationship between the log_10_ hsCRP value and leukocyte counts. Multiple comparisons were performed using ANOVA, followed by Bonferroni testing.

Multivariate analysis was carried out with the forward stepwise method of logistic regression analysis. The association between the MRI findings and the log_10_ hsCRP value was tested using logistic regression models, adjusted for age, sex, hypertension, diabetes mellitus, hyperlipidemia, renal function, metabolic syndrome, uric acid, alcohol use, and smoking. We investigated the relationship between metabolic syndrome, the hsCRP value, DWMLs, and apathy using SEM [57]. The SEM was described as path diagrams, wherein the square boxes represented measured observations and circles represented latent constructs. Single-headed arrows represented a simple regression relationship and double-headed arrows represented correlations. We examined several indices of model fit for SEM analysis (chi-square, GFI, AGFI, CFI, and RMSEA).

## Figures and Tables

**Table 1:** Characteristics of the Study Population.

	C-Reactive Protein Tertiles						*p* for Trend
	Low (*n* = 87)		Medium (*n* = 86)		High (*n* = 86)		
High-Sensitivity C-Reactive Protein	0.020–0.366 mg/L		0.379–0.982 mg/L		1.001–12.617 mg/L		
Age, mean (S.D.), years	68.8	(7.3)	67.7	(6.3)	68.8	(7.3)	NS
Male, *n* (%)	38	(43.7)	46	(53.5)	38	(44.2)	NS
Body mass index, mean (S.D.), kg/m^2^	22.5	(3.1)	23.8	(3.7)	24.2	(3.5)	0.004
Metabolic syndrome, *n* (%)	5	(5.7)	15	(17.4)	14	(16.3)	0.043
Waist circumference, mean (S.D.), cm	81.7	(7.5)	85.6	(9.9)	87.1	(9.6)	<0.001
Hypertension, *n* (%)	34	(39.1)	32	(37.2)	38	(44.2)	NS
Systolic BP, mean (S.D.), mmHg	139.8	(19.7)	139.5	(18.0)	145.2	(18.8)	0.089
Diastolic BP, mean (S.D.), mmHg	80.9	(10.9)	82.1	(10.4)	83.0	(10.9)	NS
Diabetes mellitus, *n* (%)	8	(9.2)	14	(16.3)	17	(19.8)	0.140
Hyperlipidemia, *n* (%)	24	(27.6)	32	(37.2)	30	(34.9)	NS
Ischemic heart disease, *n* (%)	4	(4.6)	9	(10.5)	1	(1.2)	0.024
Chronic kidney disease, *n* (%)	15	(17.2)	12	(14.0)	20	(23.3)	NS
Alcohol, *n* (%)	32	(36.8)	41	(47.7)	30	(34.9)	0.180
Smoking, *n* (%)	6	(7.0)	13	(15.1)	6	(7.0)	0.110
Hemoglobin A1c, mean (S.D.), %	5.56	(0.51)	5.78	(0.96)	5.74	(0.63)	0.103
LDL cholesterol, mean (S.D.), mg/dL	117.5	(29.0)	125.1	(31.4)	126.7	(38.2)	0.150
HDL cholesterol, mean (S.D.), mg/dL	73.5	(15.7)	65.2	(18.5)	63.8	(15.7)	<0.001
Triglyceridel, mean (S.D.), mg/dL	95.6	(59.7)	135.8	(107.8)	125.9	(87.9)	0.007
Uric acid, mean (S.D.), mg/dL	4.69	(1.34)	5.05	(1.21)	5.38	(1.29)	0.002
eGFR, mean (S.D.), mL/min/1.73 m^2^	74.0	(14.6)	76.1	(15.4)	71.9	(14.7)	0.183

Abbreviations: BP, blood pressure; LDL, low density lipoprotein; HDL, high density lipoprotein; eGFR, estimated glomerular filtration rate. NS, *p* > 0.2.

**Table 2:** Potential Risk Factors for Deep White Matter Lesions (DWMLs).

MRI Findings	DWMLs			Confluent DWMLs			Confluent DWMLs		
					Model 1			Model 2	
	OR	95% CI	*p*	OR	95% CI	*p*	OR	95% CI	*p*
Age, /10 years	2.456	1.647–3.663	<0.001	4.405	2.419–8.021	<0.001	5.065	2.683–9.559	<0.001
log_10_ hsCRP				3.024	1.305–7.008	0.010	2.878	1.218–6.800	0.016
Hypertension	2.620	1.508–4.553	0.001						
Metabolic syndrome *				3.211	1.141–9.003	0.027	1.996	1.228–3.243	0.005

Age, sex, hypertension, diabetes, hyperlipidemia, chronic kidney disease, metabolic syndrome, smoking, alcohol habit, uric acid, and log10hsCRP were included in the forward stepwise method of logistic regression analysis. * Model 1, the presence of metabolic syndrome; Model 2, metabolic syndrome was replaced by metabolic syndrome score.

## Supplementary Materials

Supplementary materials can be found at https://www.mdpi.com/1422-0067/20/8/1905/s1.

## Abbreviations

AGFI	adjusted goodness of fit index
ANOVA	analysis of variance
CBF	cerebral blood flow
CFI	comparative fit index
DWMLs	deep white matter lesions
eGFR	estimated glomerular filtration rate
FLAIR	fluid attenuated inversion recovery
GFI	goodness of fit index
HDL	high-density lipoprotein
hsCRP	high-sensitivity C-reactive protein
IL-1	interleukin-1
IL-1β	interleukin-1β
IL-6	interleukin-6
IL-10	interleukin-10
LDL	low-density lipoprotein
MRI	magnetic resonance imaging
mST	modified Stroop test
Ox-LDL	oxidized low-density lipoprotein
RBMT	Rivermead Behavioral Memory Test
RMSEA	root mean square error of approximation
SEM	structural equation modeling
TNF-α	tumor necrosis factor-α
