# Supplementary material for: Low-Grade Inflammation Is Associated with Apathy Indirectly via Deep White Matter Lesions in Community-Dwelling Older Adults: The Sefuri Study
Source: Int J Mol Sci. 2019 Apr 17;20(8):1905. doi: 10.3390/ijms20081905 (PMC6514652; doi:10.3390/ijms20081905)
Supplement: Supplementary file 1 [file ijms-20-01905-s001.pdf]

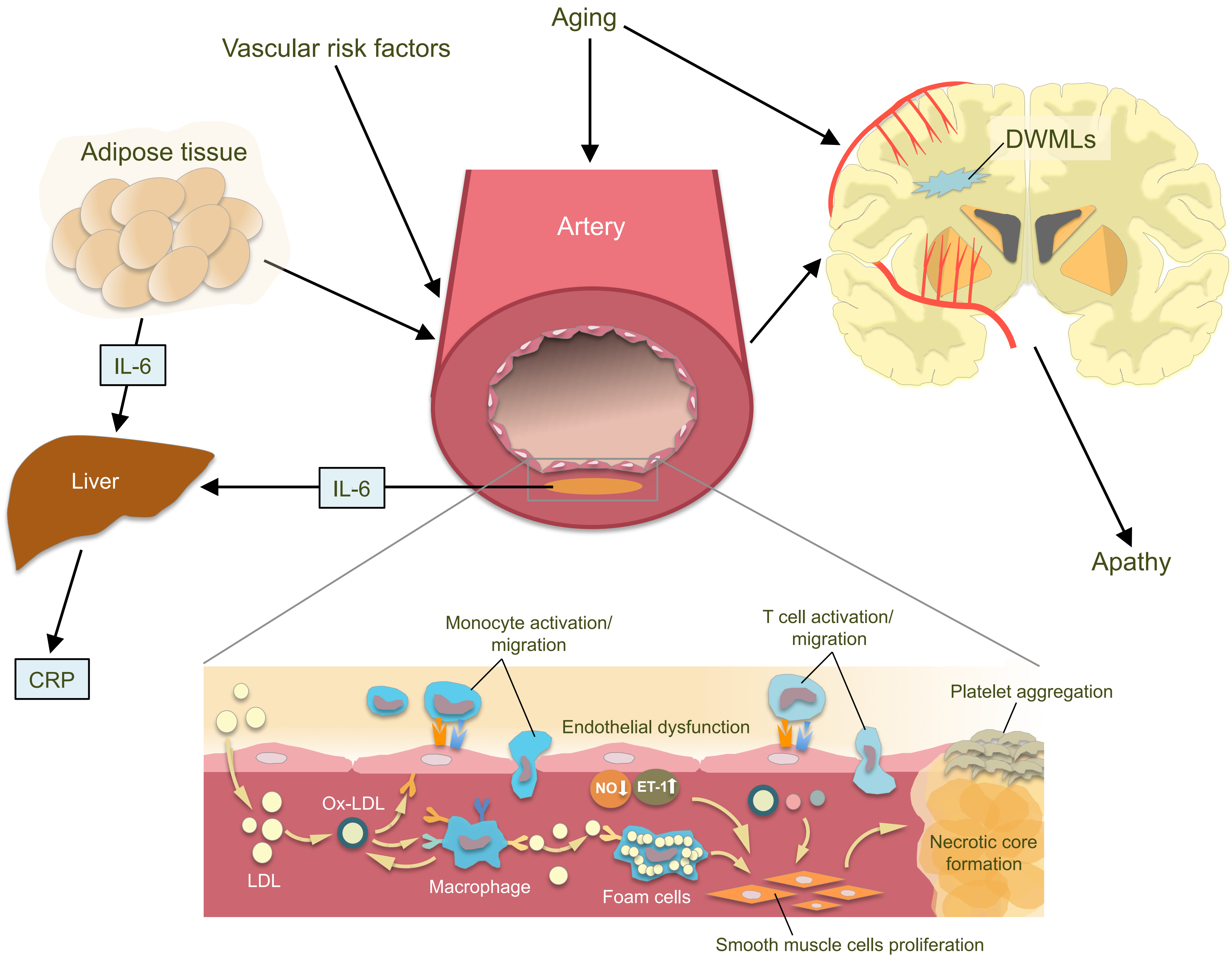

Aging
Vascular risk factors
Adipose tissue
IL-6
Liver
IL-6
CRP
Artery
DWMLs
Apathy
Monocyte activation/
migration
T cell activation/
migration
Platelet aggregation
Endothelial dysfunction
NO
ET-1
Ox-LDL
LDL
Macrophage
Foam cells
Necrotic core
formation
Smooth muscle cells proliferation

Supplementary Figure 1. Retention of low-density lipoprotein (LDL) particles in the vessel wall is the first step of atherosclerosis; when LDL particles are trapped in an artery, LDL undergoes oxidation. Oxidized LDL (Ox-LDL) induces pro-inflammatory responses via a lectin-like oxidized LDL receptor-1. Endothelial cells activated by Ox-LDL express leukocyte adhesion molecules such as intracellular adhesion molecule-1 and vascular cell adhesion molecule-1, which increase the adhesion and migration of monocytes into the arterial wall. After migration, the monocytes differentiate into macrophages and express scavenger receptors on the cell surfaces. The internalization of Ox-LDL by means of the scavenger receptors leads to the formation of lipids oxidation and the accumulation of cholesterol esters (i.e., the formation of foam cells and plaques). As plaques evolve, activated macrophages and T cell lymphocytes are involved in the advanced atherosclerotic lesion or plaque progression. Endothelial dysfunction is a primary step in atherosclerosis development as noted by increased endothelin-1 (ET-1) expression and decreased nitric oxide (NO) production. Ox-LDL also increase the expression of growth factors (e.g., platelet-derived growth factor and fibroblast growth factor) for the migration and proliferation of smooth muscle cells, leading to the thickening of plaques, formation of a necrotic core, and ultimately plaque rupture. Atherosclerosis of the cerebral arteries induces hypoperfusion in the watershed zone between the basal ganglia perforation arteries and medullary arteries, thereby causing deep white matter lesions (DWMLs). C-reactive protein (CRP) is produced by hepatocytes under the regulatory control of circulating IL-6 from atherosclerotic lesions and from adipose tissue.
